# Eco-friendly and biodegradable cellulose hydrogels produced from low cost okara: towards non-toxic flexible electronics

**DOI:** 10.1038/s41598-019-54638-5

**Published:** 2019-12-03

**Authors:** Xi Cui, Jaslyn J. L. Lee, Wei Ning Chen

**Affiliations:** 1 Interdisciplinary Graduate School, Nanyang Technological University, 50 Nanyang Avenue, Singapore, 639798 Singapore; 2 Advanced Environmental Biotechnology Centre, Nanyang Environment & Water Research Institute, Nanyang Technological University, 1 CleanTech Loop, CleanTech One, No. 06-08, Singapore, 637141 Singapore; 3 School of Chemical and Biomedical Engineering, Nanyang Technological University, 62 Nanyang Drive, Singapore, 637459 Singapore

**Keywords:** Biological techniques, Biotechnology, Health care, Engineering, Materials science

## Abstract

With increasing resource shortage and environmental pollution, it is preferable to utilize materials which are sustainable and biodegradable. Side-streams products generated from the food processing industry is one potential avenue that can be used in a wide range of applications. In this study, the food by-product okara was effectively reused for the extraction of cellulose. Then, the okara cellulose was further employed to fabricate cellulose hydrogels with favorable mechanical properties, biodegrablability, and non-cytotoxicity. The results showed that it could be biodegraded in soil within 28 days, and showed no cytotoxicity on NIH3T3 cells. As a proof of concept, a demostration of wearable and biocompatible strain sensor was achieved, which allowed a good and stable detection of human body movement behaviors. The okara-based hydrogels could provide an alternative platform for further physical and/or chemical modification towards tissue engineering, medical supplies, or smart biomimetic soft materials.

## Introduction

Hydrogels have a 3D network structure which consists of hydrophilic polymers groups that can hold large quantities of water. This structure gives them the advantageous properties including biocompatibility, super hydrophilicity, and higher water absorption capacity. Due to these properties, hydrogels have been applied in many different areas such as ionic skin^1^, tissue engineering^2^, controlled drug delivery^3,4^, controlled drug release^5^, wound dressing and healing^6^, and so on. Therefore, more and more researchers are increasing their attention towards hydrogels in recent years. Hydrogels can be classified into two categories based on their origins, including synthetic polymers and natural polymers. In comparison to synthetic hydrogels, natural hydrogels are more safe, and also possess good biocompatibility and biodegradability. Examples of natural hydrogels include protein-based hydrogels (gelatin^7^, collagen^8^), DNA-based hydrogels and polysaccharide-based hydrogels (starch^9^, hyaluronic acid^10^, chitosan^11^, alginate^12^, and cellulose^13–15^).

Okara, which is also known as soybean waste, is a byproduct from the tofu and soymilk industry. A million tonnes of okara are generated worldwide annually, which causes  significant disposal problem^16^. Most of the okara is incinerated or filled in lands, which is a waste of resources and environmentally unfriendly. To solve this problem, many researchers are exploring environmentally friendly options to valorise okara. Dry okara contains 25.4–28.4% protein, 9.3–10.9% lipid, 3.8–5.3% low molecular weight carbohydrates, and more than 50% dietary fiber, including cellulose, hemicellulose, and lignin^17^. Carbohydrates and proteins in okara have been utilized as green adhesive resins instead of petrochemical synthetic resin adhesives in fiberboard bonding^18^. Dietary fiber in okara can be added in food as functional composition to promote digestion of protein, lipid, and other nutrients^19,20^. Fermentation can be used to improve the nutritional composition of okara for celling culturing and other use^21^. Cellulose, an important polysaccharide in okara, is constitutive of the linear chain of *β*-1-4 glucosidic bond linked D-glucose. This natural structure endows it with the properties of good biocompatibility, biodegradability and the potential to be utilized in green chemical engineering, for example, hydrogels synthesis. Cellulose based hydrogels can be prepared by dissolving in an ionic compounds solvent system^22^, using low- and high-molecular-weight crosslinkers^23^, and combining with clay^24^. Okara based hydrogels had been fabricated with glutaraldehyde and poly (vinyl alcohol) by using microvave-assistant heating and exploited as a water reservoir for plant growth^25^. The okara was used without further treatment to fabricate hydrogels and the mechanical properties of the hydrogel were not discussed.

In this study, pure cellulose was obtained from okara by removing its protein and lipids. Furthermore, the hemicellulose and lignin were also removed. The extracted okara cellulose was then used to synthesize okara cellulose hydrogels by crosslinking. Physical properties, biodegradability, and cytotoxicity of okara cellulose hydrogels were tested, as well as its potential application as a wearable sensor. This study will provide an insight into the use of the food byproduct such as okara for the production of natural hydrogels.

## Materials and Methods

### Materials

Fresh okara was provided by Unicurd Food Company Pte Ltd., (Singapore) and kept in sealed plastic bags at −80 °C fridge. Sodium hydroxide (NaOH), BioXtra, ≥98%; Pectinase from *Aspergillus niger*, powder, 1.15 U/mg, sulfuric acid (H_2_SO_4_), ACS reagent, 95.0–98.0%; hydrochloric acid (HCl), ACS reagent, 37%; powdered cellulose, United States Pharmacopeia (USP) reference standard, derived from cotton linters; sigmacell cellulose, 20 μm, derived from cotton linters; anthrone, analytical standard; epichlorohydrin (ECH) ≥99%, 1.18 g/mL; lithium hydroxide monohydrate (LiOH·H_2_O), bioxtra were all purchased from Sigma-Aldrich (St. Louis, MO, USA) and without purification. NIH3T3 cells were purchased from ATCC (USA).

### Cellulose extraction from okara

The method of extracting cellulose from okara in this study was summarized and modified from previous study^26,27^. Briefly, dry okara powder was obtained from fresh okara after freeze drying for 24 h and ball milling for 1 h. 5 g prepared dry okara powder was put into reagent bottle and mixed with 20 mL deionized water. 0.05 g pectinase powder was dissolved in 0.5 mL deionized water and mixed with okara suspension, and the pH was adjusted to 3 by adding hydrochloric acid with the pH meter to indicate the pH of the suspension needed to break the cell wall of okara. The mixed suspension was kept in a water bath at 50 °C for 24 h. The suspension was washed three times with centrifuge and the supernatant was discarded. 30 mL 5% NaOH was added into the solid and the suspension was kept in a water bath at 80 °C for 3 h to remove fatty acid and hemicellulose from okara. The suspension was washed three times with centrifuge and the supernatant was discarded. 30 mL 2% surfactant was added into the solid and was kept in a water bath at 50 °C overnight to remove protein from okara. The suspension was washed three times with centrifuge and the supernatant was discarded. 10 mL 1% bleach was added into the solid and was kept in a water bath at 75 °C for 2 h to bleach the sample. The suspension was washed three times with centrifuge and the supernatant was discarded. The solid part (okara cellulose) was freeze-dried for 24 h and put into a sealed tube for further use. The quantity of cellulose in extracted sample was tested according to the methods established by Viles^28^. Briefly, 0.2 g extracted sample was digested by cold sulfuric acid (60%) for 30 minutes, the solution was measured to 10–200 mg/0.5 mL cellulose. 0.5 mL solution was added to 2 mL deionized water and cooled in room temperature. After that, 4 mL of 0.1% anthrone solution (dissolved in concentrated sulfuric acid) was added. The solution was mixed immediately and cooled at room temperature. After approximately 10 min, the solution was cooled in ice water bath. The colorimeter is adjusted to zero according to the signal of the blank and was read at 625 nm. The reference blank was prepared with 0.5 mL sulfuric acid, 2 mL deionized water and 4 mL of 0.1% anthrone solution. The referred standard was prepared with cellulose standard and adjusted to 40, 80, 120, 160, and 200 mg per 0.5 mL and the same procedure was applied. The purity of cellulose in extracted sample can be calculated by:

1 $${\rm{Purity}}=\frac{{{\rm{m}}}_{{\rm{c}}}}{{{\rm{m}}}_{{\rm{s}}}}\times 100 \% $$

m_c_ is the weight of cellulose in the extracted sample, m_s_ is the weight of extracted sample.

The yield of okara cellulose can be expressed as:

2 $${\rm{Yield}}=\frac{{m}_{s}}{{m}_{o}}\times 100 \% $$

m_s_ is the weight of extracted sample, m_0_ is the weight of dried okara powder.

### Synthesis of okara cellulose hydrogels

The solution for dissolving okara cellulose was prepared by LiOH, urea, and water at the weight ratio of 8:15:77 according to Lina Zhang’s work^29,30^. The okara cellulose was added rapidly with stirring (1000 rpm) at 25 °C to achieve a solution with a concentration of 6% wt after the prepared solution was precooled. Bubbles and impurities were removed from the solution with centrifuge. The okara cellulose hydrogels were synthesized by adding different amounts of epichlorohydrin (molar ratio of epichlorohydrin (ECH) to the anhydroglucose unit (AGU) is 0.5, 0.6, 0.7, 0.8, and 0.9, respectively) into the cellulose solution. The solution was stirred to mix and kept in an ice water bath for 2 h to obtain a pre-crosslinked solution. After that, the solution was poured into hydrogel molds carefully and kept in 4 °C fridge overnight. The hydrogels were taken out from the molds carefully and immersed in water. The water was replaced three times a day for one week to remove LiOH, urea, unreacted cellulose, epichlorohydrin and other impurities in the synthesized okara cellulose hydrogels. The cotton cellulose hydrogels were prepared with the same method.

### Swelling property of okara cellulose hydrogels

The swelling properties of okara cellulose hydrogels at different ECH-to-AGU molar ratio were measured. The hydrogels were immersed in water to swell by absorbing water until balance at 25 °C. The weight of the hydrogels in this condition is m_wet_. The swollen hydrogels were dried in a vacuum oven at 80 °C until balance. The weight of hydrogels in this condition is m_dry_. The swelling properties (water content) of hydrogels can be expressed as:

3 $${{\rm{C}}}_{{\rm{H}}2{\rm{O}}}=\frac{{{\rm{m}}}_{{\rm{wet}}}-{{\rm{m}}}_{{\rm{dry}}}}{{{\rm{m}}}_{{\rm{wet}}}}\times 100 \% $$

m_wet_ is the weight of swollen hydrogels after balance, and m_dry_ is the weight of absolute dried hydrogels.

Okara cellulose hydrogels at different ECH-to-AGU molar ratios were all tested for three times.

### Characterization of okara cellulose hydrogels

#### Fourier-transform infrared spectroscopy (FTIR) analysis

Fourier-transform infrared spectroscopy was measured on a PerkinElmer Spectrum One FTIR. The freeze-dried samples were milled to powder and mixed with KBr. The mixed powder were pressed to pieces. The analysis was operated by scanning scale of 4000–400 cm^−1^ for 32 times.

#### Field emission scanning electron microscope (FESEM) analysis

The okara cellulose hydrogels and cotton cellulose hydrogels were immersed in liquid nitrogen to freeze, broken quickly to get the fracture surface, followed by freeze-drying overnight before testing. The freeze-dried hydrogels and powder samples of okara powder, okara cellulose, and cotton cellulose were adhered to the silicon wafer and were sputtered with platinum. Samples were analyzed by a field emission scanning electron microscope (JSM6710-FESEM).

### Mechanical property of okara cellulose hydrogels

Stretching property of okara cellulose hydrogels were tested by Mechanical Tester MTS C42. Hydrogel sheets of 30 mm * 5 mm were tested at the speed of 2 mm/min. Cyclic test of okara hydrogel at molar ratio of ECH-to-AGU of 0.7 was done from maximum stretching strain 10% to 80% in sequence. The Young’s modulus was calculated from the stress-strain curves.

### Cytotoxicity of okara cellulose hydrogels

Okara hydrogel pieces (100 mg/piece) were sterilized by UV light and the sterile hydrogels was immersed in 20 mL Dulbecco’s Modified Eagle Medium (DMEM) for 20 days. The hydrogel extracted DMEM was diluted to 25% and 50%. The NIH3T3 cells were seeded in 96-well plates at 37 °C before the cytotoxicity test. The cells were incubated with different concentrations (0%, 25%, 50%, 100%) of hydrogel treated DMEM for 72 h. After that, 3-(4,5-dimethylthiazol-2-yl)-2,5-diphenyltetrazolium bromide (MTT) assays were performed following the standard protocols^31^. The absorbance of the solution in MTT assay was read with microplate reader at 570 nm to measure the Optical Density (OD) value. The cell viability was evaluated by MTT assay with the cells cultured with normal DMEM as control and calculated by:

4 $${\rm{Viability}}=\frac{{{\rm{OD}}}_{{\rm{treatment}}}}{{{\rm{OD}}}_{{\rm{contorl}}}}\times 100 \% $$

OD_treatment_ is the OD values of samples treated with hydrogel extracted DMEM, OD_control_ is the average of OD values of samples treated with normal DMEM.

All the tests were repeated five times, with standard deviation represented by error bars for the cell viability percentages measured.

### Biodegradation of okara cellulose hydrogels

Natural soil was obtained from NTU campus and used as the biodegradation environment. Okara cellulose hydrogels (5 × 10 mm^2^) were buried approximately 10 cm in depth from the surface of the soil. The average incubation temperature is 25 °C. The degraded hydrogels fragments were taken out one by one after 2 to 30 days of burying. The weight of degraded hydrogels fragments was measured by balance.

### Okara cellulose hydrogels applied as a wearable sensor

The okara cellulose hydrogels were applied as a wearable sensor in this study. The two ends of okara cellulose hydrogel were connected to an electrochemical workstation via the wires. The wires were immersed into the pre-crosslinking solution during crosslinking process and connected to the hydrogels naturally when the hydrogels were synthesized. The signal of electric current was recorded by electrochemical workstation while the okara cellulose hydrogel was stretched to the different length. The okara cellulose hydrogel was put on the surface of the wrist and stretched while the wrist bending to the angle of 30°, 60°, and 90°. The electric current signal was read and recorded while the wrist bending. A similar test was done on the surface of the finger.

### Ethical approval and informed consent for human subjects

Ethical approval was granted by Nanyang Technological University, under the Institutional Review Board, Singapore and research was performed according to guidelines. The Declaration of Helsinki on ethical principles for research also guided the study. Informed written consent were taken from the volunteers.

## Results and Discussion

### Cellulose extraction from okara

**Figure 1:**
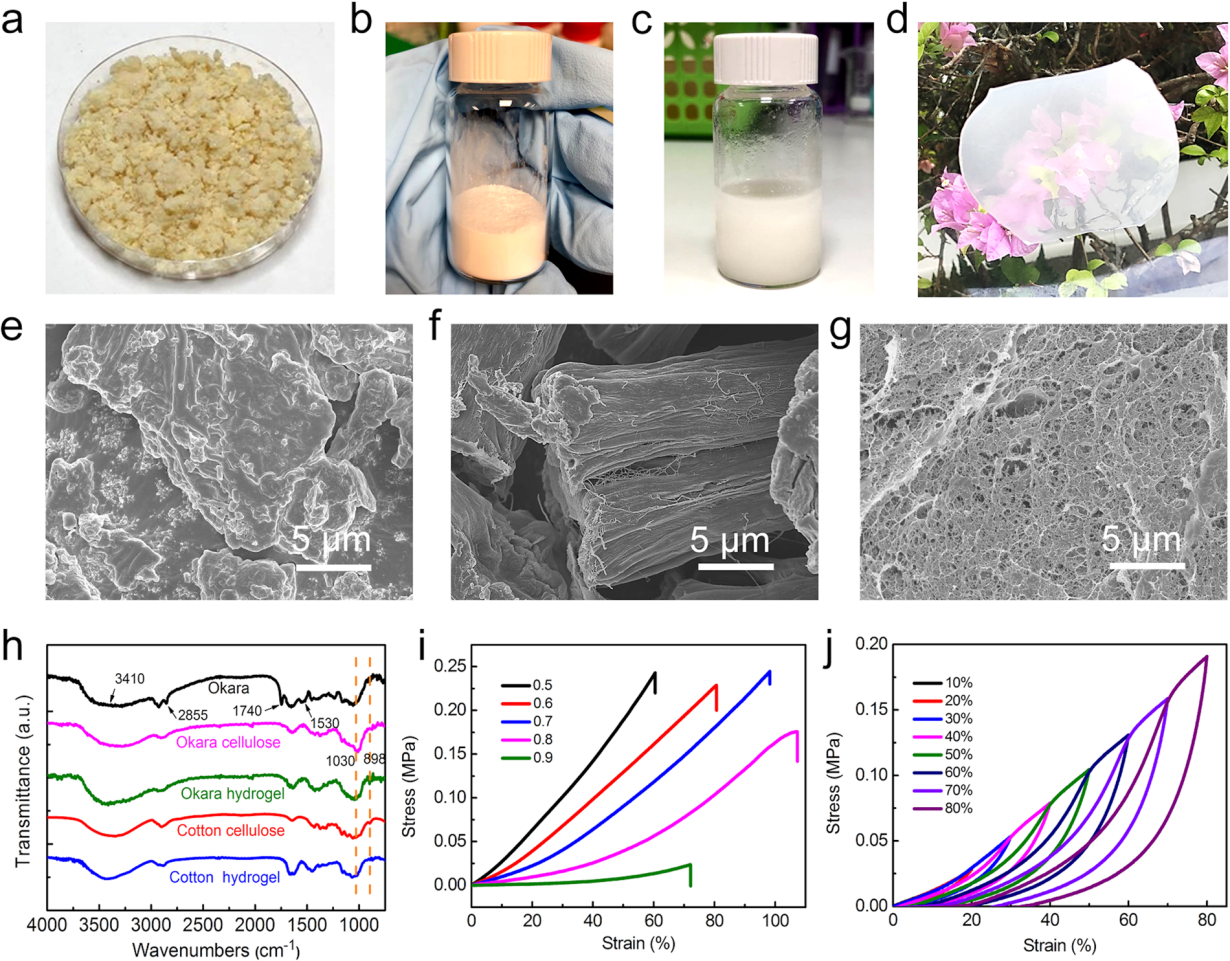
(**a**) Fresh okara, (**b**) extracted okara cellulose, (**c**) okara cellulose solution, (**d**) okara cellulose hydrogel. Field emission scanning electron microscopy image for (**e**) dried okara power, (**f**) extracted okara cellulose, (**g**) fracture surface of okara cellulose hydrogel. (**h**) Fourier-transform infrared spectroscopy (FTIR) spectra of okara powder, okara cellulose, okara cellulose hydrogel, sigmacell cotton cellulose, and cotton cellulose hydrogel. (**i**) Tensile stress-strain curves of the okara cellulose hydrogels at different ECH-to-AGU molar ratios under tension. (**j**) Tensile stress-strain curves of the okara cellulose hydrogel (molar ratio of ECH-to-AGU 0.7) during cycles with various maximum stretching.

Fresh okara (Fig. 1a) was pre-treated by freeze drying and ball milling. The obtained fine powdered okara (Fig. S1a) was treated with the followed extraction steps. The substances in okara were easier to be separated and released after ball milling as the cell wall were broken. Lipid and hemicellulose were hydrolysed and removed by alkali. Protein in okara was removed by reacting with surfactant. The sample was oxidized and bleached by strong oxidant to remove lignin and other impurities. After cell wall degrading, lipid, hemicellulose, protein, and lignin removing, cellulose (Fig. 1b) was extracted from okara with the purity of 91% and the yield of 8.8%. The appearance of extracted okara cellulose powder was similar to that of cotton cellulose powder (Fig. S1b). Okara cellulose powder was dissolved to form cellulose solution (Fig. 1c), and the appearance of the synthesized okara cellulose hydrogel is shown (Fig. 1d). The cost of our detergent method is $120 per kilogram of okara, which is much cheaper than that of the enzyme method ($27,000). Furthermore, the other chemicals used in the extraction method were all commonly used chemicals. The unfavourable effects on the environment is expected to be offset by the use of mature technologies on a larger scale, which will not be discussed in this work.

### Swelling property of okara cellulose hydrogels

Swelling property of okara cellulose hydrogels was tested according to the weight change of okara cellulose hydrogels before and after drying and summarized in Table 1. When the molar ratio of epichlorohydrin (ECH) to the anhydroglucose unit (AGU) of cellulose increased from 0.5 to 0.9, the water content of okara cellulose hydrogels increased from 88% to 94%, gradually. The results indicated that the more crosslinker was added, the more water the hydrogels could hold, which might be attributed to the higher density of the crosslinking network.

**Table 1:** Water content and mechanical properties of okara cellulose hydrogels.

Sample	M, ECH:AGU	C_H2O_/wt%	σ/MPa	ε/%	E/MPa
1	0.5	88 ± 2	0.243	60.5	0.37
2	0.6	89 ± 2	0.229	80.6	0.28
3	0.7	91 ± 3	0.245	98.2	0.12
4	0.8	92 ± 1	0.176	107	0.066
5	0.9	94 ± 2	0.024	72.1	0.0087

M, molar ratio of ECH to AGU of okara hydrogels; C_H2O_, water content of the okara cellulose hydrogels; σ, ε, and E are stress at break, break strain, and Young’s modulus under tension.

### FTIR analysis

FTIR characterization of okara powder, okara cellulose, okara cellulose hydrogels, sigmacell cotton cellulose, and cotton cellulose hydrogels are shown in Fig. 1h. All of these samples have the same absorption at 3410 cm^−1^, which is attributed to –OH stretching vibrations. The signal at 2855 cm^−1^ in okara sample is assigned to –CH_3_ group, which was not found in okara cellulose. The signal at 1744 cm^−1^ in okara sample is assigned to C=O group, which was also not found in okara cellulose. The signal at −1530 cm^−1^ is C–C stretches in the aromatic ring, which was also not found in okara cellulose. These three signals were attributed to the lipid, protein, hemicellulose, and lignin in okara. The spectra of okara cellulose and cotton cellulose shared almost the same absorption band. These results indicated that these impurities were removed during the extraction step and the obtained okara cellulose was pure. The peak at 898 cm^−1^ is characteristic signal of the *β*-1-4-glycosidic bond between glucose units in cellulose. The absorption band at 1030 cm^−1^ is assigned to –C–O– group. The ratio of signal intensity of –C–O– (1060 cm^−1^) to that of *β*-glycosidic linkage (898 cm^−1^) of okara cellulose hydrogels is larger than that of okara cellulose. This verified that the quantity of –C–O– group increased after crosslinking and the hydrogels were synthesized successfully^32^. This increase could also be found when cotton cellulose was compared to cotton cellulose hydrogels.

### Morphology of okara, okara cellulose and okara cellulose hydrogels

The morphology of okara powder, okara cellulose, okara cellulose hydrogels, cotton cellulose, and cotton cellulose hydrogels was characterized by field emission scanning electron microscopy (FESEM). From the FESEM results, agglomerates were found intertwined with each other in the okara samples (Fig. 1e). This made it hard to identify the different substances. After removing the impurities, wirelike fibers could be observed clearly in the okara cellulose (Fig. 1f). This demonstrated that the purity of cellulose increased after removing impurities. The fiber of extracted okara cellulose was finer than that of the cotton cellulose (Fig. S1c). A porous structure was found on the fracture surface of okara cellulose hydrogels (Fig. 1g). This was the crosslinked structure of okara cellulose, as a result of reacting with the crosslinker. The crosslinked structure of okara cellulose hydrogels was observed to be denser than that of cotton cellulose hydrogels (Fig. S1d).

### Mechanical property of okara cellulose hydrogels

Tensile tests were used to investigate the stretching property of okara cellulose hydrogels. (Fig. 1i,j) Okara cellulose hydrogels at different ECH-to-AGU molar ratio were prepared by adding different amount of crosslinker into the cellulose solution. The mechanical properties of okara hydrogels are summarized in Table 1. The moduli of the cellulose hydrogels decreased regularly with the increasing molar ratio of ECH-to-AGU, as a result of the increase in the cross-link density amongst cellulose molecule chains. The highest tensile strength at fracture of the okara cellulose hydrogels was 0.245 MPa when the crosslinking ECH-to-AGU molar ratio was 0.7. The highest tensile strain at fracture of the okara cellulose hydrogels was 107% when the crosslinking ECH-to-AGU molar ratio was 0.8. (Fig. 1i) The highest tensile strain (107%) of okara cellulose hydrogel was larger than that of cotton cellulose hydrogel (53%) with only chemical crosslinking^32^, indicating that the okara cellulose hydrogel had good tensile property. Due to the hydrogen bonds in the cellulose chains and the circumvoluted network of cellulose chains, the okara cellulose hydrogels exhibited a strain-hardening behavior. The cyclic test of the okara cellulose hydrogel (ratio of 0.7) under the tensile test were carried out and hysteresis and tiny irreversible stretch of the hydrogel were observed. (Fig. 1j) This phenomenon in okara cellulose hydrogel was similar to that of the cotton cellulose hydrogel^32^. Okara cellulose hydrogel at ECH-to-AGU molar ratio of 0.7 was selected for further characterization and application, due to its superior mechanical property as compared to the others.

### Cytotoxicity Test by MTT assay

**Figure 2:**
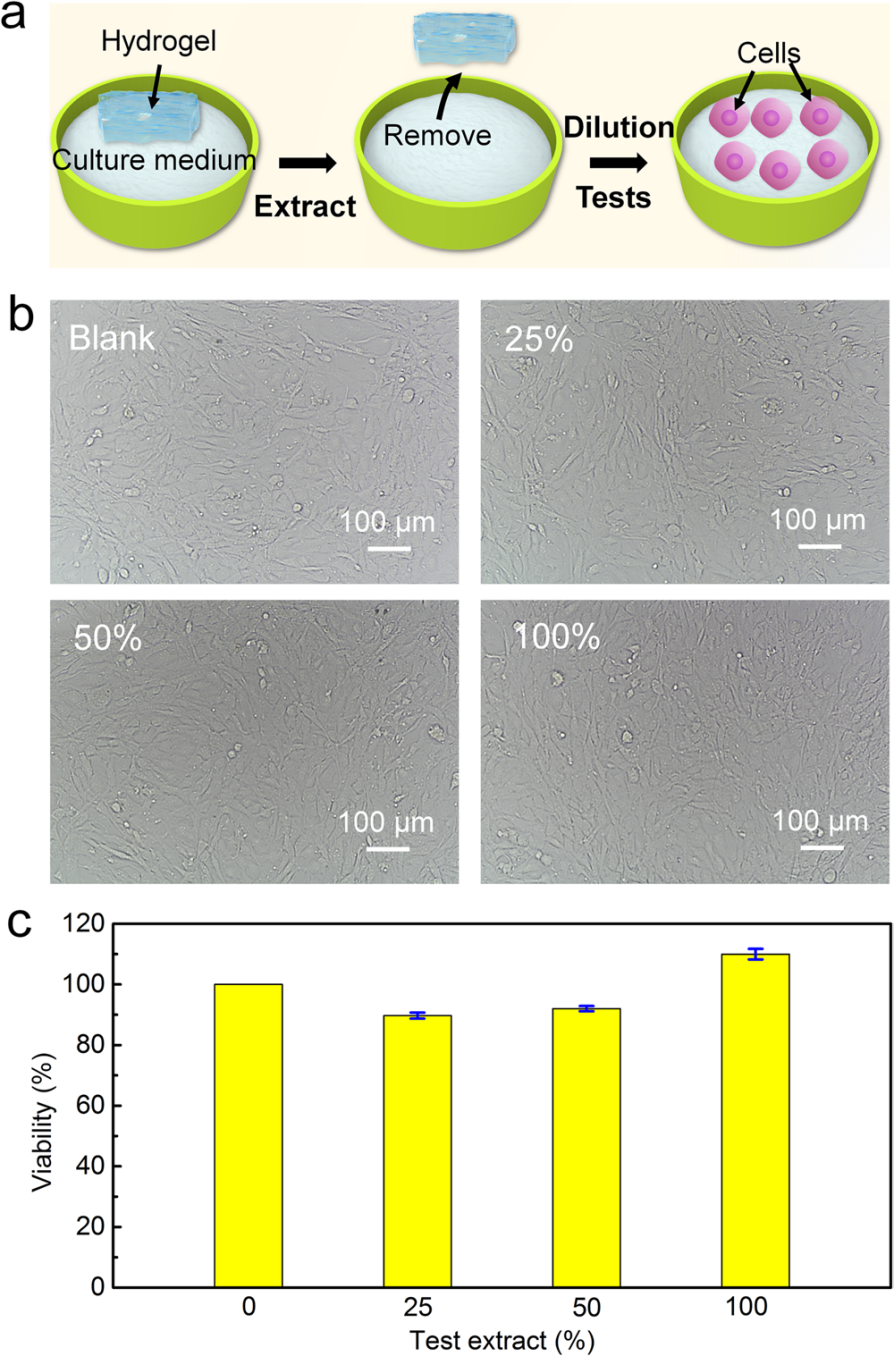
(**a**) Scheme of cytotoxicity testing of okara cellulose hydrogel extracts on NIH3T3 cells by MTT assay. (**b**) Optical micrographs of NIH3T3 cells exposed to 100%, 50%, and 25% of hydrogel extract, and blank. (Magnification: ×200). (**c**) Viability of NIH3T3 cells after incubating with hydrogel extracted DMEM at different concentration.

The cytotoxicity of okara cellulose hydrogels was analyzed by the MTT assay (Fig. 2). The morphologies of NIH3T3 cells exposed to 100%, 50% and 25% of hydrogel extracted DMEM solution were similar to that of control (DMEM without hydrogel extract) in optical micrographs (Fig. 2b**)**. The viability values of NIH3T3 cells after incubation with hydrogel extracted DMEM at different concentrations are summarized in Fig. 2c. The viability values of NIH3T3 cells were 109.9 ± 1.72% for 100% okara hydrogel extracted DMEM, while 92 ± 0.86% for 50% okara hydrogel extracted DMEM and 89.7 ± 0.99% for 25% okara hydrogel extracted DMEM. The cytotoxicity results are similar to the study reported by Peng^15^, which indicate that 100%, 50%, and 25% of hydrogel extracted DMEM have no cytotoxicity on NIH3T3 cells according to MTT assay. The lack of cytotoxicity of the okara cellulose hydrogels are attributed to the natural cellulose matrix and the major chemical components of the hydrogel extract are only a small number of residual reagents such as LiOH, urea, and unreacted epichlorohydrin after washing by water for one week. Some other wearable sensors made of synthetic polymers based hydrogels showed uncertain cytotoxicity^1,33^.

### Biodegradation test

**Figure 3:**
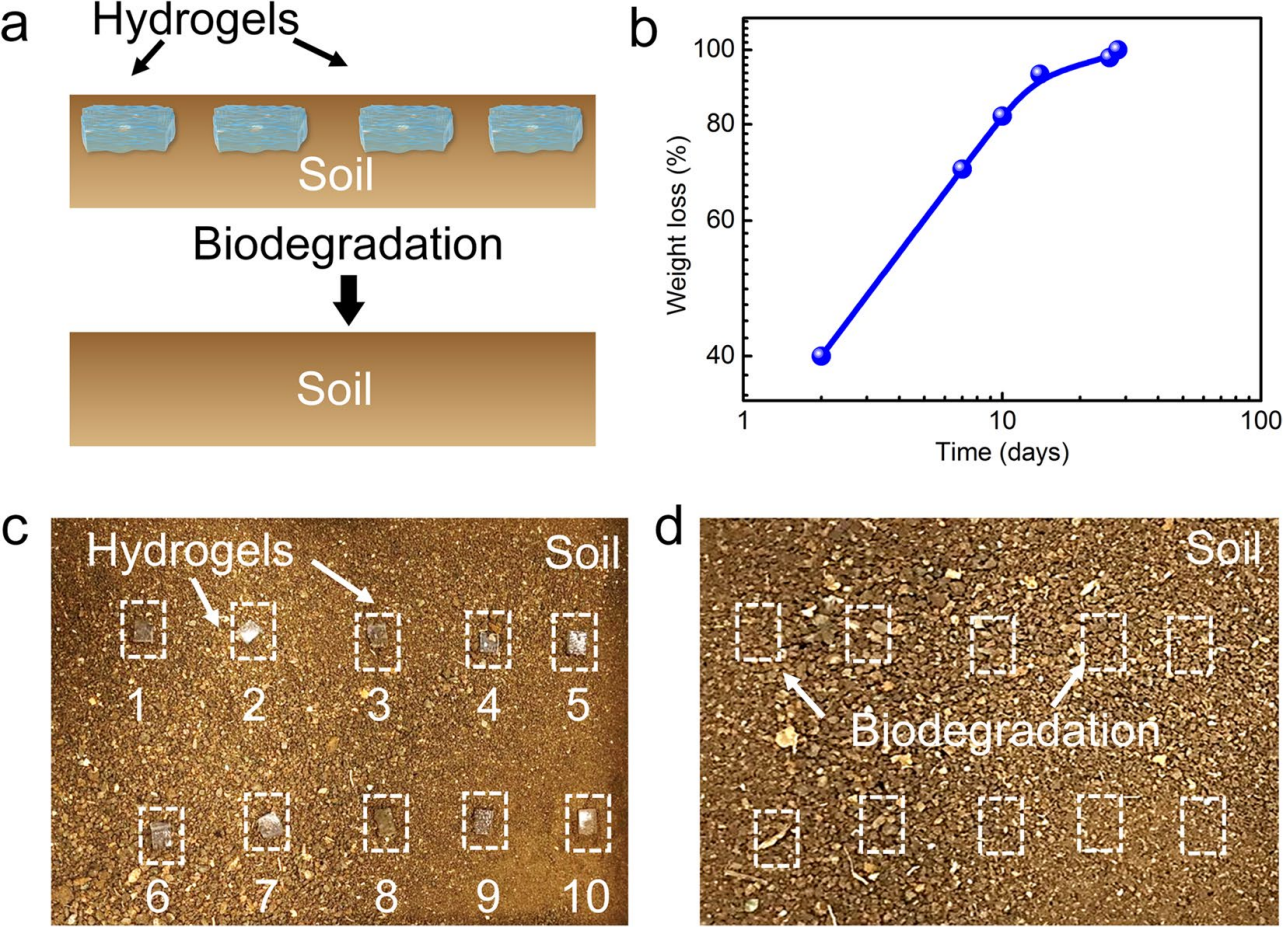
(**a**) Scheme of biodegradation test for okara cellulose hydrogels. (**b**) Weight loss of okara hydrogels during degradation time. Pieces of okara cellulose hydrogels in the soil on the first day (**c**) and day 28 (**d**) in the biodegradable test.

The biodegradability of okara cellulose hydrogels was evaluated by the soil test. (Fig. 3) Fig. 3a shows the scheme of the biodegradation test, the hydrogels in soil before and after degradation. Ten pieces of hydrogels were buried into the soil on the first day. (Fig. 3c) The weight of okara cellulose hydrogels lost during the degradation time buried in the soil are shown in Fig. 3b. The hydrogels lost 40% of their original weight within the initial two days, mainly due to the evaporation of water from the hydrogels. More than 80% of hydrogels weight was lost after 14 days. After the hydrogels were buried into the soil for 26 days, only small fragments of the hydrogels could be found, and the weight loss reached up to 97%. The hydrogels were observed to be completely degraded after 28 days of burial in the soil (Fig. 3d). Li^34^ reported that starch hydrogel could be degraded by α-amylase within a few hours, but no obvious degradation of starch hydrogel was detected without the existence of amylase. Similar to the degradation of starch hydrogel by α-amylase, the synthesized okara cellulose hydrogels could be biodegraded by normal soil (without adding enzyme) within one month, which implied good biodegradability of okara cellulose hydrogel. The biodegradation of the hydrogels was caused by the microorganisms in soil^35^ and the hydrogels were stable in normal environment without specific enzyme or microorganism.

### Okara cellulose hydrogel used as wearable sensor

**Figure 4:**
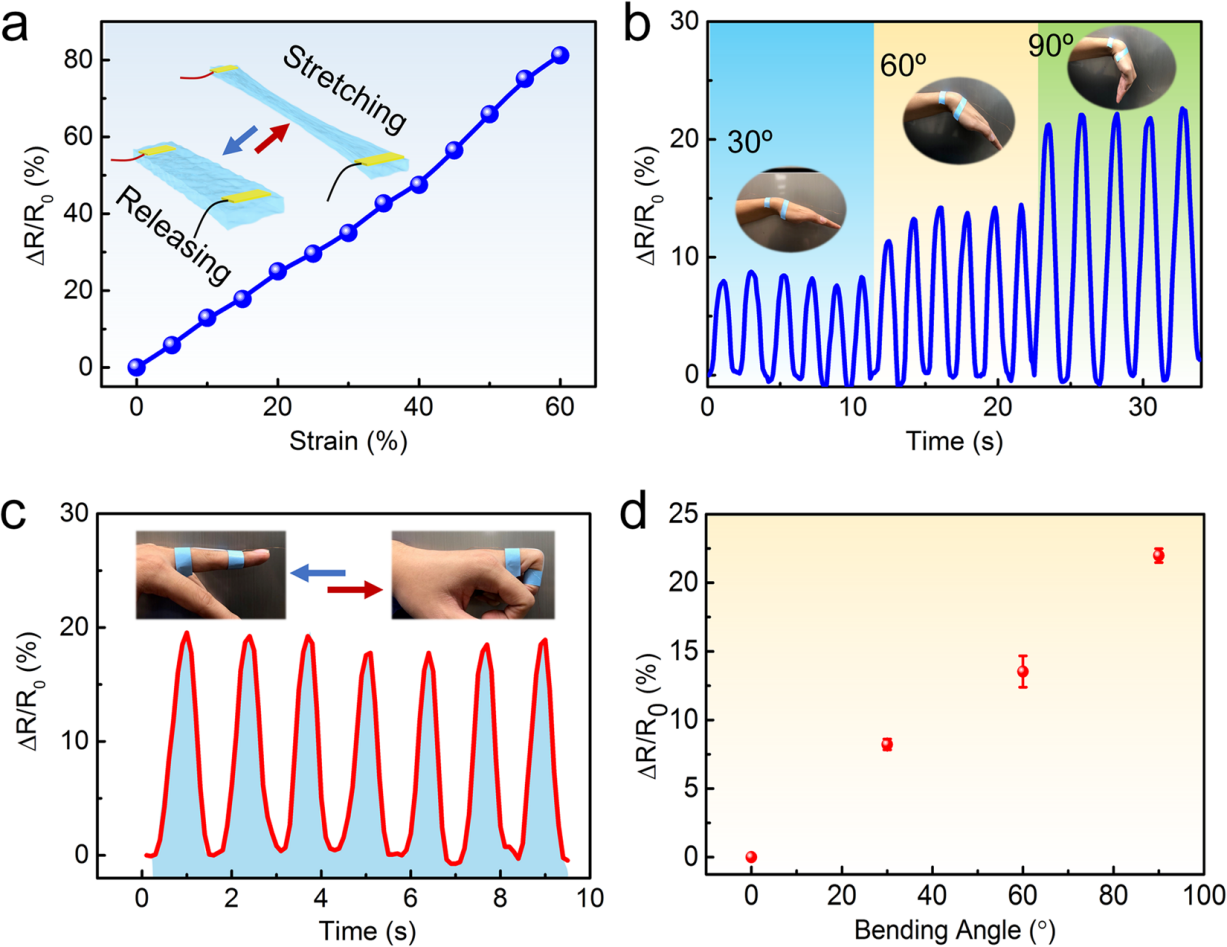
(**a**) Resistance change ratio of okara cellulose hydrogel with tensile strain from 0% to 60%. (**b**) Resistance change ratio of okara hydrogel sensors on wrist bending to the angle of 30°, 60°, 90° and summarized in **(d**). (**c**) Resistance change ratio of okara cellulose hydrogel on finger bending.

The resistance of okara cellulose hydrogel changed while it was stretched and pressed. The resistance change ratio could be defined as the ratio of the resistance change (ΔR = R − R_0_) to the initial resistance R_0_, described as ΔR/R_0_. The relationship between resistance change ratio (ΔR/R_0_) and tensile strain is shown in Fig. 4a. The resistance change ratio increased linearly when the strain increased in the range from 0% to 60%. This phenomenon might be caused by the density decrease of the hydrogel crosslinking structure while stretching. Furthermore, the okara cellulose hydrogel was used as a sensor directly touched on the skin, to detect the movement of the wrist and the finger, as shown in Fig. 4b,c. The okara cellulose hydrogel was contacted on the surface of the wrist and stretched while the wrist was bending to 30°, 60° and 90° (Fig. 4b). The signal of electric current was read and recorded by electrochemical workstation while the wrist was bending. The resistance change ratio of bending angel 30°, 60° and 90° was 8.22%,13.53%, 21.98%, respectively (Fig. 4d). A similar test was conducted on the surface of the finger (Fig. 4c). The resistance change ratio can reach 20% with the finger bending to 90°. The electric performance of the okara cellulose hydrogel was stable as cycle number increased in this study. Only small varies between cycles were caused by finger movement, as the finger could not move exactly to the same position in every cycle. The water contained in okara cellulose hydrogels will evaporate slowly, which may affect the stability of the sensors, even though this phenomenon was not found in our study. However, okara cellulose hydrogels have the potential to be used as wearable conductive devices, according to the results.

## Conclusion

In this study, natural cellulose was extracted form okara by removing protein, lipids, hemicellulose, and lignin. Okara cellulose hydrogels were synthesized by reacting okara cellulose with the crosslinker. The structure of okara cellulose hydrogels were characterized by Fourier-transform infrared spectroscopy and field emission scanning electron microscope. The swelling property and tensile property of okara cellulose hydrogels were tested. These results indicated that the prepared okara cellulose hydrogels have good tensile property, good biodegradability with no cytotoxicity, and sensitive electric signal. The okara cellulose hydrogels were applied as a wearable sensor in this study. This is of great significance in the side-stream product in food processing and the potential application in drug delivery, tissue engineering, and biosensors.

## Supplementary information

Supplementary information

## Data Availability

All datasets generated are available from corresponding author on reasonable request.
