# Supplementary material for: Eco-friendly and biodegradable cellulose hydrogels produced from low cost okara: towards non-toxic flexible electronics
Source: Sci Rep. 2019 Dec 3;9:18166. doi: 10.1038/s41598-019-54638-5 (PMC6890720; doi:10.1038/s41598-019-54638-5)
Supplement: Supplementary file 1 — Supplementary information [file 41598_2019_54638_MOESM1_ESM.pdf]

Supplementary Information

Eco-friendly and biodegradable cellulose hydrogels produced from low cost okara: towards non-toxic flexible electronics

Xi Cui, Jaslyn J.L. Lee, Wei Ning Chen*

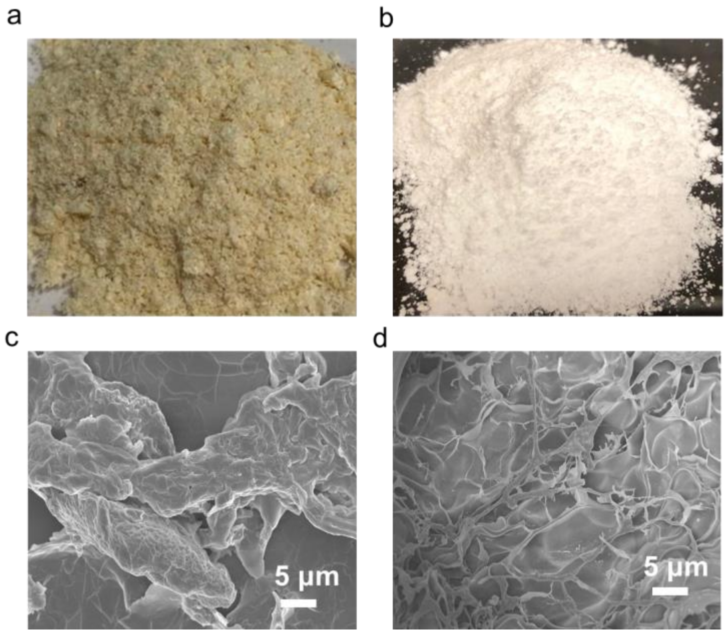

Fig. S1. (a) Dried okara powder, (b) sigmacell cotton cellulose. FESEM image of (c) sigmacell cotton cellulose and (d) sigmacell cotton cellulose hydrogel.
